# The combined treatment of Molnupiravir and Favipiravir results in a potentiation of antiviral efficacy in a SARS-CoV-2 hamster infection model

**DOI:** 10.1016/j.ebiom.2021.103595

**Published:** 2021-09-24

**Authors:** Rana Abdelnabi, Caroline S. Foo, Suzanne J.F. Kaptein, Xin Zhang, Thuc Nguyen Dan Do, Lana Langendries, Laura Vangeel, Judith Breuer, Juanita Pang, Rachel Williams, Valentijn Vergote, Elisabeth Heylen, Pieter Leyssen, Kai Dallmeier, Lotte Coelmont, Arnab K. Chatterjee, Raf Mols, Patrick Augustijns, Steven De Jonghe, Dirk Jochmans, Birgit Weynand, Johan Neyts

**Affiliations:** a KU Leuven Department of Microbiology, Immunology and Transplantation, Rega Institute for Medical Research, Laboratory of Virology and Chemotherapy, B-3000 Leuven, Belgium; b UCL Institute of Child Health, 30 Guilford Street, London, WC1N 1EH, United Kingdom; c Calibr at Scripps Research, La Jolla, CA 92037, USA; d KU Leuven, Department of Pharmaceutical and Pharmacological Sciences, Drug Delivery & Disposition, Box 921, 3000 Leuven, Belgium; e KU Leuven Department of Imaging and Pathology, Translational Cell and Tissue Research, Division of Translational Cell and Tissue Research, B-3000 Leuven, Belgium; f Global Virus Network, GVN, Baltimore, MD 21201, USA

**Keywords:** SARS-CoV-2, Antivirals, Molnupiravir, Favipiravir, hamsters, coronavirus

## Abstract

**Background:**

Favipiravir and Molnupiravir, orally available antivirals, have been reported to exert antiviral activity against SARS-CoV-2. First efficacy data have been recently reported in COVID-19 patients.

**Methods:**

We here report on the combined antiviral effect of both drugs in a SARS-CoV-2 Syrian hamster infection model. The infected hamsters were treated twice daily with the vehicle (the control group) or a suboptimal dose of each compound or a combination of both compounds.

**Findings:**

When animals were treated with a combination of suboptimal doses of Molnupiravir and Favipiravir at the time of infection, a marked combined potency at endpoint is observed. Infectious virus titers in the lungs of animals treated with the combination are reduced by ∼5 log10 and infectious virus are no longer detected in the lungs of >60% of treated animals. When start of treatment was delayed with one day a reduction of titers in the lungs of 2.4 log10 was achieved. Moreover, treatment of infected animals nearly completely prevented transmission to co-housed untreated sentinels. Both drugs result in an increased mutation frequency of the remaining viral RNA recovered from the lungs of treated animals. In the combo-treated hamsters, an increased frequency of C-to-T mutations in the viral RNA is observed as compared to the single treatment groups which may explain the pronounced antiviral potency of the combination. Interpretation: Our findings may lay the basis for the design of clinical studies to test the efficacy of the combination of Molnupiravir/Favipiravir in the treatment of COVID-19. Funding: stated in the acknowledgment.

**Research in Context**

**Evidence before this study**

We reported earlier that the influenza drug Favipiravir results in a marked protective activity against SARS-CoV-2 infection in the hamster model. Recently, Favipiravir (as Avifavir) was reported to results in a faster virological response, a shorter time to clinical symptoms and reduced mortality rates. Also Molnupiravir has been reported to inhibit SARS-CoV-2 infection in vitro and in vivo. Recent data from a phase 2 trial with Molnupiravir showed a reduction in the time required to reach negative isolation of infectious virus from nasopharyngeal swabs from participants with symptomatic SARS-CoV-2 infection.

**Added value of this study**

This study reports on the potent antiviral effect of the combination of Molnupiravir and Favipiravir in the SARS-CoV-2 hamster infection model. Whereas suboptimal doses of either drug result respectively in ∼1.2 log_10_ reduction in infectious virus titers in the lungs, the combination causes a >4.5 log_10_ reduction of such titers. When start of the combination treatment was delayed for 6 or 24 h, a reduction of infectious titers in the lungs of respectively 3.1 and 2.4 log_10_ was achieved. Moreover, treatment of infected animals nearly completely prevented transmission to co-housed untreated sentinels. We also demonstrate that the potent antiviral efficacy in the combination group may be explained by an increased mutation rate of the viral genome as compared to the monotherapy treatment

**Implications of all the available evidence**

This study provides strong evidence that a combined all-oral treatment of Molnupiravir and Favipiravir should be explored for the treatment of SARS-CoV-2 infections.

Alt-text: Unlabelled box

## 1 Introduction

The severe acute respiratory syndrome coronavirus 2 (SARS-CoV-2) was first identified in Wuhan, China in December 2019 [1]. Since then, the virus rapidly spread around the globe with more than 177 million cases and 3.8 million deaths reported until 15^th^ June 2021 [www.COVID-19.who.int]. Infection with SARS-CoV-2 results in coronavirus-induced disease (COVID-19) which is characterized by a wide range of symptoms including fever, dry cough, muscle and/or joint pain, headache, decreased sense of taste and smell and diarrhea. The disease can progress into severe complications such as acute respiratory distress syndrome (ARDS), respiratory failure, septic shock as well as multi-organ failure, which are mainly attributed to a massive cytokine storm and exaggerated immune response [2].

To date, there are no approved, selective coronavirus antivirals to treat or prevent infections. Even those vaccinated may not all be protected against infection and disease, in particular following infection with variants that are less susceptible to the current vaccines. Antivirals against SARS-CoV-2 will, at least when given early enough after a positive test or after onset of symptoms, reduce the chance to progress to (more) severe disease. In addition, such antiviral drugs will be useful to protect for example healthcare workers and high-risk patients in a prophylactic setting. Such drugs are also needed for the treatment of immunodeficient patients who do not mount a (sufficiently robust) immune response following vaccination. Since the de novo development and approval of (a) specific, highly potent antiviral(s) for SARS-CoV-2 may require years, the main focus for COVID-19 treatment in the current pandemic is to repurpose drugs that have been approved or in clinical trials for other diseases [3].

We recently demonstrated that the anti-influenza drug Favipiravir results in a pronounced antiviral activity in SARS-CoV-2-infected hamsters [4]. The efficacy of the drug is currently being explored in multiple phase II clinical studies. Interim data from a multicenter, open-labeled study of Avifavir (Favipiravir) in patients with COVID-19, reveals a faster virological response, a shorter time to clinical symptoms and reduced mortality rates compared to patients receiving the Standard of Care [5]. The ribonucleoside analogue, N4-hydroxycytidine (NHC, EIDD-1931), was initially developed as an influenza inhibitor, but exerts also broader-spectrum antiviral activity against multiple viruses belonging to different families of RNA viruses. Activity against SARS-CoV-2 has been reported in cell lines and primary human airway epithelial cell cultures [6].

Both Favipiravir and NHC act through lethal mutagenesis. Incorporation into viral RNA results in the accumulation of deleterious transition mutations beyond a permissible error threshold to sustain the virus population, leading to error catastrophe [7,8]. The orally bioavailable, pro-drug counterpart of NHC [9], Molnupiravir (EIDD-2801, MK-4482) was shown to result in an antiviral effect against SARS-CoV-2 in a Syrian hamster model [10], in a mouse model [11] and in ferrets [12]. Data from a first-in-human, phase 1, randomized, double-blind, placebo-controlled study in healthy volunteers indicate that the drug is well tolerated and that plasma exposures exceed the expected efficacious doses based on scaling from animal models [13]. The drug is currently being assessed for its potential as an antiviral treatment of SARS-CoV-2 infection in Phase 2 clinical trials of infected patients (NCT04405570, NCT04405739). Recent interim data (on one secondary objective) were reported in 202 non-hospitalized adults who had signs or symptoms of COVID-19 and active confirmed SARS-CoV-2 infection. At day 5, a reduction in positive viral culture from nasopharyngeal swabs was noted in subjects who received molnupiravir (0/47) as compared to placebo (6/25). No safety signals were identified in the 202 participants [14].

We here report on the pronounced combined activity of Favipiravir and Molnupiravir in the hamster infection model.

## 2 Methods

### 2.1 SARS-CoV-2

The SARS-CoV-2 strain used in this study, BetaCov/Belgium/GHB-03021/2020 (EPI ISL 109 407976|2020-02-03), was recovered from a nasopharyngeal swab taken from an RT-qPCR confirmed asymptomatic patient who returned from Wuhan, China in the beginning of February 2020. A close relation with the prototypic Wuhan-Hu-1 2019-nCoV (GenBank accession 112 number MN908947.3) strain was confirmed by phylogenetic analysis. Infectious virus was isolated by serial passaging on Huh7 and Vero E6 cells [4]; passage 6 virus was used for the study described here. Live virus-related work was conducted in the high-containment A3 and BSL3+ facilities of the KU Leuven Rega Institute (3CAPS) under licenses AMV 30112018 SBB 219 2018 0892 and AMV 23102017 SBB 219 20170589 according to institutional guidelines.

### 2.2 Cells

Vero E6 cells (African green monkey kidney, ATCC CRL-1586) were cultured in minimal essential medium (MEM, Gibco) supplemented with 10% fetal bovine serum (Integro), 1% non-essential amino acids (NEAA, Gibco), 1% L- glutamine (Gibco) and 1% bicarbonate (Gibco). End-point titrations on Vero E6 cells were performed with medium containing 2% fetal bovine serum instead of 10%. Huh7 cells were cultured in Dulbecco's modified Eagle's medium (DMEM, Gibco) supplemented with 10% fetal bovine serum (Integro), 1% bicarbonate (Gibco), 2% HEPES buffer (Gibco). Both cells were kept in a humidified 5% CO_2_ incubator at 37°C.

### 2.3 Compounds

For the first pilot experiment, EIDD-2801 was kindly provided by Calibr at Scripps Research (USA). For further studies, Molnupiravir (EIDD-2801) was purchased from Excenen Pharmatech Co., Ltd (China) and was formulated as 50 mg/ml stock in a vehicle containing 10% PEG400 (Sigma) and 2.5% Kolliphor-EL (Sigma) in water. Favipiravir was purchased from BOC Sciences (USA) and was formulated as a 50 mg/mL stock in 3% sodium bicarbonate (Sigma) in water.

### 2.4 SARS-CoV-2 infection model in hamsters

The hamster infection model of SARS-CoV-2 has been described before [4,15]. Female Syrian hamsters (Mesocricetus auratus) were purchased from Janvier Laboratories and kept per two in individually ventilated isolator cages (IsoCage N Bio-containment System, Tecniplast) at 21°C, 55% humidity and 12:12 day/night cycles. Housing conditions and experimental procedures were approved by the ethics committee of animal experimentation of KU Leuven (license P065-2020). For infection, female hamsters of 6-8 weeks old were anesthetized with ketamine/xylazine/atropine and inoculated intranasally with 50 µL containing 2×10^6^ TCID50 SARS-CoV-2 (day 0). On day 4 post-infection, animals were euthanized for sampling of the lungs and further analysis by i.p. injection of 500 μL Dolethal (200 mg/mL sodium pentobarbital). No randomization methods were used and confounders were not controlled, though all caretakers and technicians were blinded to group allocation in the animal facility, and to sample numbers for analysis (qPCR, titration, histology and deep sequencing). In addition, when the animals arrived at the animal facility they were ear-tagged and allocated as pairs (i.e. without any selection criteria) into cages then the cages were assigned into different treatment groups again without any preference. Since all animals had the same age and roughly of the same weight; all are females, we believe there is no a real confounder to be considered in the study that may impact the outcome of the experiments.

### 2.5 Treatment regimen

For combination therapy, hamsters were treated starting from day0 just before infection with SARS-CoV-2 or at 6h post-infection (pi) or starting from day1 pi with the vehicle (control group), 150 mg/kg EIDD-2801 (oral gavage) and 300 mg/kg Favipiravir (intraperitoneal, i.p.) twice daily. All the treatments continued until day 3 pi. Hamsters were monitored for appearance, behavior and weight. Since Molnupiravir was given orally, we had to administer favipiravir by i.p. injection due to the limitation of the total volume to be given by oral gavage to hamsters (not >5ml/kg). Adminstration of higher volumes by oral gavage results in an overfilling of the stomach resulting in insufficient food intake and hence weight loss. For this reason one of the drugs (Favipiravir) was given by injection to avoid this volume limitation of the oral gavage. At day 4 pi, hamsters were euthanized by i.p. injection of 500 μL Dolethal (200mg/mL sodium pentobarbital, Vétoquinol SA). Lungs were collected and viral RNA and infectious virus were quantified by RT-qPCR and end-point virus titration, respectively.

### 2.6 Transmission study

Two groups of index hamsters were infected with SARS-CoV-2 and treated with either vehicle or the combined Molnupiravir and Favipiravir therapy (150+300 mg/kg) twice daily starting from day0. On day1 pi (6 hours after the first dose), each index hamster was co-housed with a contact hamster (non-infected, non-treated hamsters) in one cage and the co-housing continued until the sacrifice day at 2.5 days after start of exposure. The treatment of index hamsters was continued until day3 pi. At day 4 pi, both index and contact hamsters were euthanized as mentioned before and lungs were collected to assess viral loads.

### 2.7 SARS-CoV-2 RT-qPCR

Hamster lung tissues were collected after sacrifice and were homogenized using bead disruption (Precellys) in 350 µL TRK lysis buffer (E.Z.N.A.® Total RNA Kit, Omega Bio-tek) and centrifuged (10.000 rpm, 5 min) to pellet the cell debris. RNA was extracted according to the manufacturer's instructions. RT-qPCR was performed on a LightCycler96 platform (Roche) using the iTaq Universal Probes One-Step RT-qPCR kit (BioRad) with N2 primers and probes targeting the nucleocapsid [15]. Standards of SARS-CoV-2 cDNA (IDT) were used to express viral genome copies per mg tissue [4].

### 2.8 End-point virus titrations

Lung tissues were homogenized using bead disruption (Precellys) in 350 µL minimal essential medium and centrifuged (10,000 rpm, 5min, 4°C) to pellet the cell debris. To quantify infectious SARS-CoV-2 particles, endpoint titrations were performed on confluent Vero E6 cells in 96- well plates. Viral titers were calculated by the Reed and Muench method [16] using the Lindenbach calculator and were expressed as 50% tissue culture infectious dose (TCID_50_) per mg tissue.

### 2.9 Histology

For histological examination, the lungs were fixed overnight in 4% formaldehyde and embedded in paraffin. Tissue sections (5 μm) were analyzed after staining with hematoxylin and eosin and scored blindly for lung damage by an expert pathologist. The scored parameters, to which a cumulative score of 1 to 3 was attributed, were the following: congestion, intra-alveolar hemorrhagic, apoptotic bodies in bronchus wall, necrotizing bronchiolitis, perivascular edema, bronchopneumonia, perivascular inflammation, peribronchial inflammation and vasculitis.

### 2.10 Deep sequencing and analysis of whole genome sequences

Genomic sequences from all samples were obtained using SureSelect^XT^ target enrichment and Illumina sequencing. Reads generated were trimmed with Trim Galore (https://github.com/FelixKrueger/TrimGalore). Duplicated reads were removed using Picard (http://broadinstitute.github.io/picard). Reads from the inoculation sample were mapped to the SARS-CoV-2 reference genome (NC_045512) from GenBank using BWA-MEM [17]. The mapping quality was checked using Qualimap and the consensus whole genome sequence was generated using QUASR [18,19]. Reads from the lung samples were mapped to this unique reference sequence. Genomes with less than less than a 100 read depth were excluded. Variants above 1% and with a minimum of 2 supporting reads per strand were identified at sites with a read depth of ≥ 10 using VarScan [20].

### 2.11 Pharmacokinetics analysis of compounds in plasma

Plasma samples were analyzed after protein precipitation with methanol for favipiravir and with acetonitrile for EIDD-1931 (containing the internal standard nelfinavir) by RP-HPLC with MS-MS detection (Acquity H-class UPLC, Waters, Milford, MA, USA and Xevo TQ-S micro Waters, Milford, MA, USA). For Favipiravir, separation was performed using a Kinetex XB - C18 column (2.6 μm, 2.1 × 50 mm; Phenomenex, Utrecht, the Netherlands) held at 40 °C. Methanol (solvent A) and 0.05% formic acid in water (solvent B) were used as eluents at 500 µL/min. Gradient elution was performed as follows: 5% of solvent A during 1 min, increase of solvent A to 90% in 0.5 min, 90% of solvent A during 1 min, decrease of solvent A to 5% in 0.5 min and 5 % of solvent A during 2 minutes to re-equilibrate the column prior to the next injection. MS/MS was carried out with an HESI source in negative ionization mode for favipiravir and in positive ionization mode for nelfinavir on a Xevo TQ-S micro mass detector (Waters, Milford, MA, USA). The mass transitions used for the detection of the different compounds were: m/z favipiravir 156.00→113.00, m/z nelfinavir 568.15→330.10. In case of EIDD-1931, separation was performed using an Acquity UPLC BEH Amide column (1.7 μm, 2.1 × 50 mm; Waters, Milford, MA, USA) held at 40 °C. Acetonitrile (solvent A) and 0.1% formic acid in water (solvent B) were used as eluents at 500 µL/min. Elution was performed in an isocratic way with 80% solvent A and 20% solvent B for 3 minutes. An MS/MS positive ionization mode was carried out with an HESI source on a Xevo TQ-S micro mass detector (Waters, Milford, MA, USA). The mass transitions used for the detection of the different compounds were: m/z nelfinavir 568.15→330.10, m/z EIDD-1931 260.00→127.90. Calibration curves were made on the day of analysis by serial dilution in plasma.

### 2.12 Sample size justification

For antiviral efficacy, we want to detect at least 1 log_10_ reduction in viral RNA levels in treated subjects compared to the untreated, infected control group. Group size was calculated on the independent t-test with an effect size of 2.0 and a power of 80% (effect size = deltamean/SD = 1 log_10_ decrease in viral RNA/0.5 log_10_), resulting in 5-6 animals/group. Sample sizes maximized considering limits in BSL3 housing capacity, numbers of animals that can be handled under BSL3 conditions, and availability of compounds.

### 2.13 Statistics

The detailed statistical comparisons, the number of animals and independent experiments that were performed is indicated in the legends to figures. “Independednt experiments” means that experiments were repeated separately on different days. The analysis of histopathology was done blindly. All statistical analyses were performed using GraphPad Prism 9 software (GraphPad, San Diego, CA, USA). Statistical significance was determined using the non-parametric Mann Whitney U-test. P-values of <0.05 were considered significant. No adjustment was done for multipule testing.

### 2.14 Ethics

Housing conditions and experimental procedures were done with the approval and under the guidelines of the ethics committee of animal experimentation of KU Leuven (license P065-2020).

### 2.15 Data Availability

All of the data generated or analyzed during this study are included in this published article.

### 2.16 Role of funding source

The Funders had no role in study design, data collection, data analyses, interpretation, or writing of report.

## 3 Results

### 3.1 The combined Molnupiravir/Favipiravir treatment enhances the in vivo efficacy

To study the combined efficacy of Molnupiravir and Favipiravir in the hamster infection model, we selected a suboptimal dose for each compound that results in ≤2 log10 reduction in the lung infectious virus titers (TCID_50_/mg lung tissue). Based on our own studies [4], a dose of 300 mg/kg (BID) was selected for Favipiravir. For Molnupiravir (EIDD-2801), we first evaluated the (dose-response) effect of the compound in SARS-CoV-2-infected hamsters to select an appropriate suboptimal dose for the combination study. In this pilot study, 6-8 weeks female SG hamsters were treated orally with Molnupiravir (either 75, 150, or 200 mg/kg, BID) or the vehicle (i.e. the control group) for four consecutive days starting one hour before infection with SARS-CoV2. At day four post-infection (pi), the animals were euthanized and lungs were collected for quantification of infectious virus titers. Molnupiravir treatment resulted in a dose-dependent reduction in the infectious virus titers in the lungs whereas the intermediate and high doses, but not the 75 mg/kg dose BID, significantly reduced infectious virus lung titers (supplementary Fig. S1). The reduction in infectious virus titers (TCID_50_/mg tissue) in the lungs of hamsters treated BID with 150 and 200 mg/kg was 1.9 (P=0.0015) and 3.5 (P=0.008) log_10_, respectively (supplementary Fig. S1). Based on these results, we selected the 150 mg/kg (BID) dose of Molnupiravir for the combination study.

**Fig. 1:**
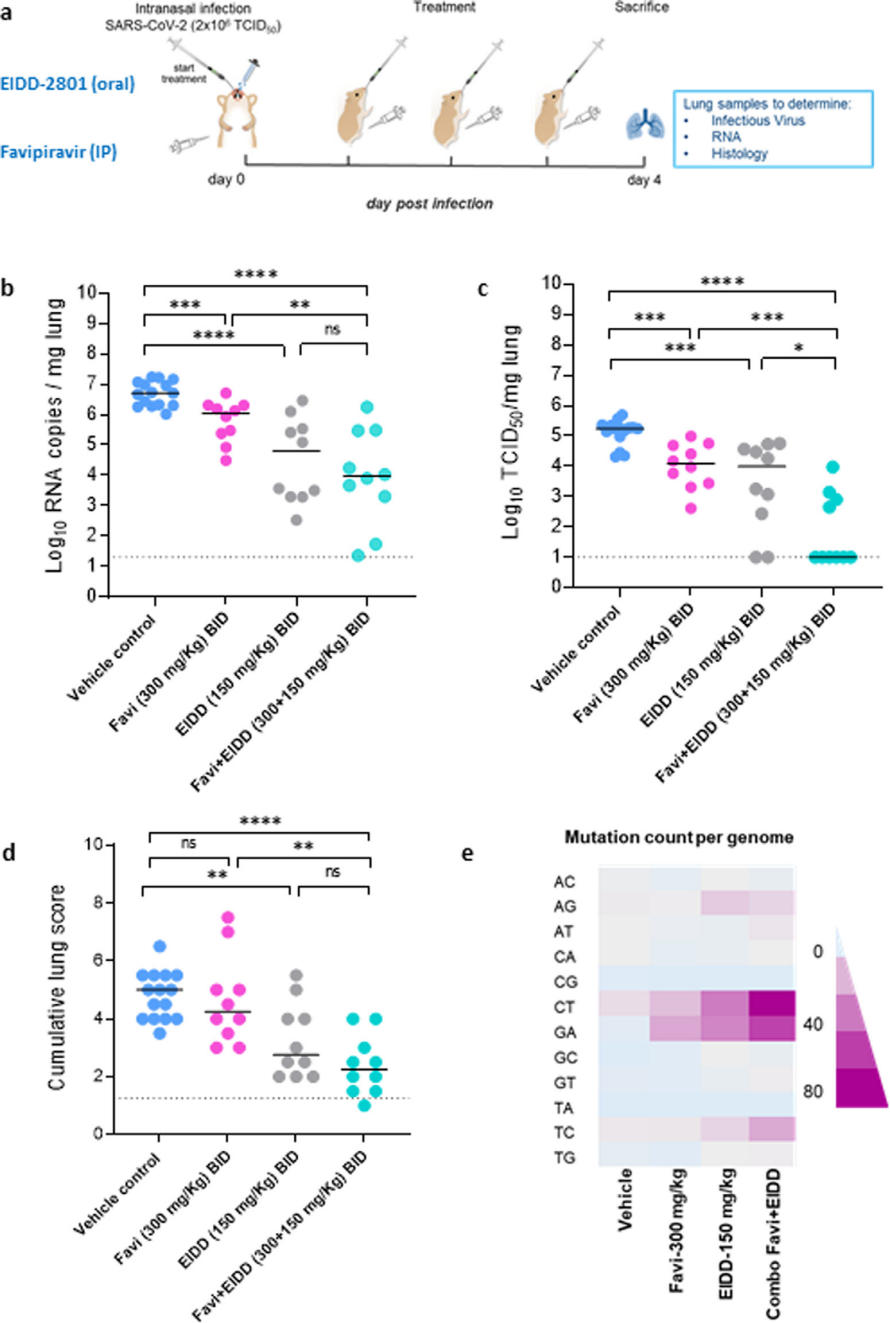
Combined efficacy of Favipiravir and Molnupiravir (EIDD-2801) against SARS-CoV-2 in a hamster infection model. (a) Set-up of the study. (b) Viral RNA levels in the lungs of control (vehicle-treated), Favipiravir-treated (300 mg/kg, BID), EIDD-2801-treated (150 mg/kg, BID) and combination-treated (Favipiravir+EIDD-2801 at 300+150 mg/kg, BID, respectively) SARS-CoV-2−infected hamsters at day 4 post-infection (pi) are expressed as log_10_ SARS-CoV-2 RNA copies per mg lung tissue. Individual data and median values are presented. (c) Infectious viral loads in the lungs of control (vehicle-treated), Favipiravir-treated, EIDD-2801-treated and combination-treated (Favipiravir+EIDD-2801) SARS-CoV-2−infected hamsters at day 4 pi are expressed as log_10_ TCID_50_ per mg lung tissue. Individual data and median values are presented. (d) Cumulative severity score from H&E stained slides of lungs from control (vehicle-treated), Favipiravir-treated, EIDD-2801-treated and combination-treated (Favipiravir+EIDD-2801) SARS-CoV-2−infected hamsters. Individual data and median values are presented and the dotted line represents the median score of untreated non-infected hamsters. (e) Mean mutation count (per the whole genome) in the viral RNA isolated from the lungs of control (vehicle-treated), Favipiravir-treated (300 mg/kg, BID), EIDD-2801-treated (150 mg/kg, BID) and combination-treated (Favipiravir+EIDD-2801 at 300+150 mg/kg, BID, respectively) SARS-CoV-2−infected hamsters at day 4 post-infection (pi). Data were analyzed with the Mann−Whitney U test. *P < 0.05, **P < 0.01, ***P < 0.001, ****P < 0.0001, ns=non-significant. Favi=Favipiravir, EIDD=EIDD-2801. All data (panels B, C, D) are from two independent experiments with 15, 10, 10 and 10 animals for respectively the vehicle, Favipiravir 300 mg/kg, EIDD-2801 150 mg/kg and Favipiravir+EIDD-2801 condition.

Next, we studied what the combined efficacy is of these suboptimal doses of Molnupiravir and Favipiravir (Fig. 1A). Briefly hamsters were treated with the vehicle (i.e. the control group) or Favipiravir (300 mg/kg, BID) or Molnupiravir (150 mg/kg, BID) or the combination of Favipiravir and Molnupiravir (at 300+150 mg/kg (BID) respectively) for four consecutive days starting one hour before intranasal infection with SARS-CoV-2. At day four post-infection (pi), the animals were euthanized and lungs were collected for quantification of viral RNA, infectious virus titers and lung histopathology as described before [4] (Fig. 1A). Treatment with Favipiravir alone (300 mg/kg, BID) reduced viral RNA and infectious virus loads in the lungs of infected animals by 0.7 (P=0.0009) and 1.2 (P=0.0002) log_10_/mg tissue, respectively (Fig. 1B/C). Treatment with Molnupiravir (150 mg/kg BID) alone resulted in 1.9 (P<0.0001) and 1.3 (P=0.0002) log_10_/mg reduction in viral RNA and infectious virus loads respectively (Fig. 1B/C). The combined treatment resulted in a reduction of 2.7 log_10_ of lung viral RNA titers (Fig. 1B), but interestingly, in a markedly enhanced reduction in infectious virus titers (>4.5 log_10_ TCID_50_ per mg lung, P=0.02, P=0.0005 as compared to Molnupiravir and Favipiravir alone, respectively) (Fig. 1C). The reduction in infectious virus titers in the combination-treated group was also statistically significant (P=0.03) compared to the animals treated only with the higher dose of Molnupiravir (200 mg/kg, BID) for which only a 3log_10_ reduction was observed (Supplementary Fig. S2). Notably, there was no detectable infectious virus in the lungs of six out of ten hamsters in the combined treatment group (Fig. 1C). A marked improvement in the histological lung pathology scores was also observed in the combined treatment group as compared to the single favipiravir treatment, P=0.001 (Fig. 1D, Supplementary Fig. S3, Supplementary Table S1). Although the difference is non-significant, there is a trend that the combination (median histopathology score of 2.25) is better than the single molnupiravir treatment group (median histopathology score of 3), Fig. 1D, Supplementary Fig. S3, Supplementary Table S1. No significant weight loss or toxicity signs were observed during the whole treatment period (Supplementary Fig. S4). To determine the exposure of treated hamsters to Molnupiravir and Favipiravir, plasma trough levels of both compounds were measured at the time of euthanasia (16h after the last treatment), (Supplementary Table S2). The IP treatment with 300 mg/kg Favipiravir (BID) resulted in average plasma concentrations of 5.9 and 5.3 µM in the single and combination treatment groups, respectively (Supplementary Table S2). These Favipiravir plasma concentrations are matching with the ones obtained in our previous favipiravir study in this hamster model [4]. Since Molnupiravir is a prodrug which is, after absorption, rapidly converted into its active metabolite EIDD-1931 [10], we quantified this metabolite in the plasma samples at the same time point as for Favipiravir. Very low concentration of EIDD-1931 were detected in the plasma samples (average of 37 and 78 nM in the single and combination treatment groups, respectively), which is most likely attributed to the fact that the activation of Molnupiravir occurs mainly in tissues [10].

In vitro, the active metabolite of molnupiravir (EIDD-1931) resulted in potent antiviral efficacy with EC_50_ of 0.3 and 0.4 µM in Vero E6-GFP and Huh7 cells, respectively (Supplementary Fig. S5 and S6). Since favipiravir does not elicit in vitro antiviral activity against SARS-CoV2 (most likely because of inefficient metabolization to the active metabolite in the cell lines used, (Supplementary Fig. S5 and S6), it was not possible, unlike the situation in hamsters, to detect a potentiation of the activity of Molnupiravir by Favipiravir in infected cell cultures. Data on the combined in vitro activity of EIDD-1931 (the active metabolite of molnupiravir) and favipiravir either in Vero E6-GFP or Huh7 cells are presented as Supplementary Fig. S5 and S6.

Molnupiravir is known to increase the mutation frequency of MERS-CoV viral RNA in infected mice [6]. To test whether this is also the case in SARS-CoV-2-infected hamsters, we used Illumina deep sequencing to determine the SARS-CoV-2 mutations rate in remaining viral RNA in lung samples of hamsters after treatment. The Molnupiravir (150 mg/kg)/Favipiravir (300 mg/kg) combination resulted in an overall increase in the total mutation count in comparison to the single dose groups (Fig. 1E, Supplementary Table S3). More specifically, the number of C-to-T mutations (average count 68 for the combination treatment) was markedly higher as compared to the single dose groups [150 mg/kg Molnupiravir group (average count 33) and 300 mg/kg Favipiravir (average count 14) (Fig. 1E, Supplementary Table S3). These results may at least partially explain the markedly enhanced reduction in infectious viral loads observed in the combination treatment group.

### 3.2 Efficacy of the combined Molnupiravir/Favipiravir treatment in therapeutic settings

**Fig. 2:**
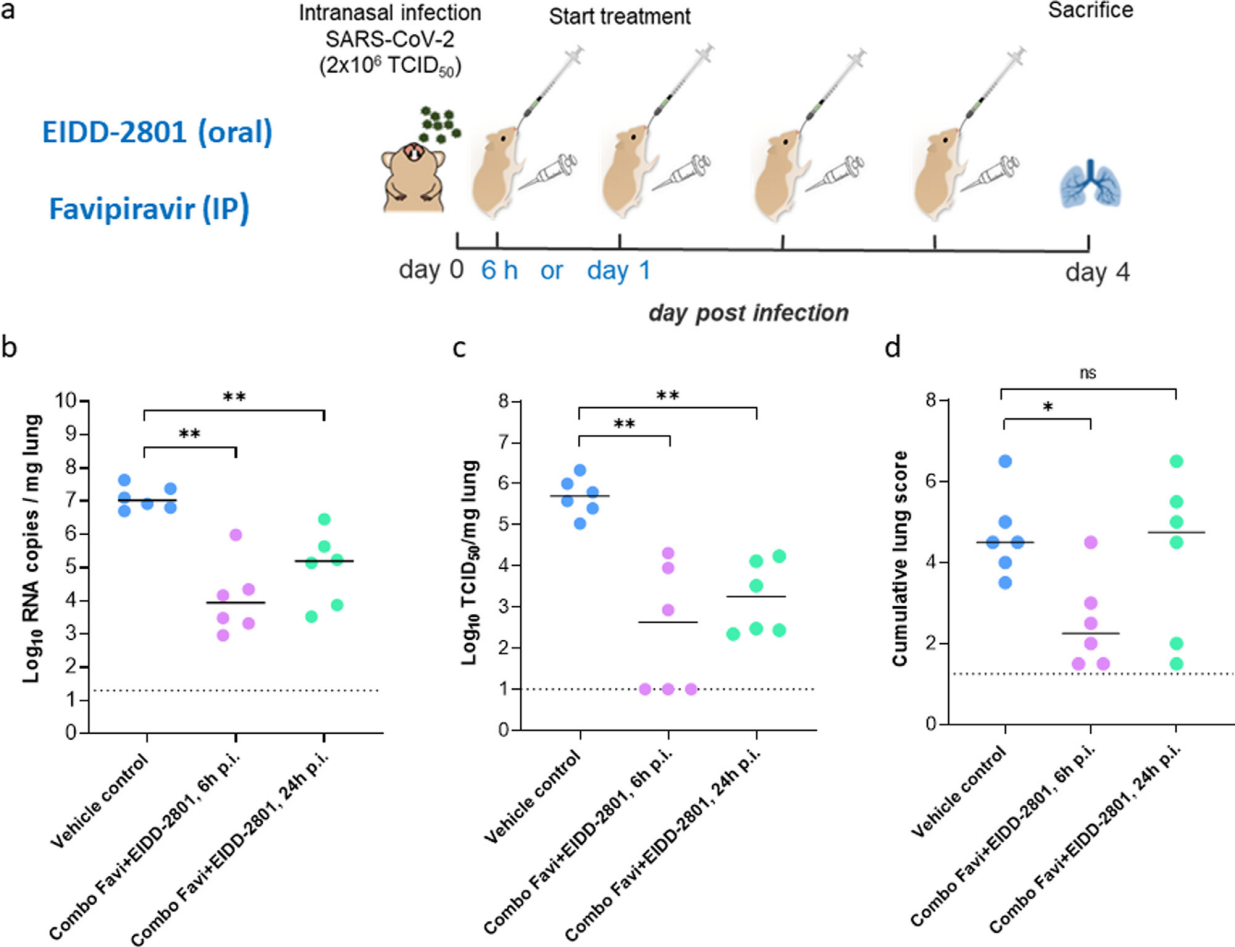
Efficacy of the combined Molnupiravir/Favipiravir treatment in therapeutic settings. (a) Set-up of the study. (b) Viral RNA levels in the lungs of control (vehicle-treated) and combination-treated (Favipiravir+EIDD-2801 at 300+150 mg/kg, BID, respectively, starting at 6h or 24h post-infection) SARS-CoV-2−infected hamsters at day 4 post-infection (pi) are expressed as log_10_ SARS-CoV-2 RNA copies per mg lung tissue. Individual data and median values are presented. (c) Infectious viral loads in the lungs of control (vehicle-treated) and combination-treated (Favipiravir+EIDD-2801 starting at 6h or 24h pi) SARS-CoV-2−infected hamsters at day 4 pi are expressed as log_10_ TCID_50_ per mg lung tissue. Individual data and median values are presented. (d) Cumulative severity score from H&E stained slides of lungs from control (vehicle-treated) and combination-treated (Favipiravir+EIDD-2801 starting at 6h or 24h pi) SARS-CoV-2−infected hamsters. Individual data and median values are presented and the dotted line represents the median score of untreated non-infected hamsters. Data were analyzed with the Mann−Whitney U test. **P < 0.01. Favi=Favipiravir, EIDD=EIDD-2801. The data are from a single experiment and with 6 animals per group.

It was next explored whether also a delayed start of treatment with the Molnupiravir/Favipiravir combination results in an antiviral effect. To that end therapy was initiated either at 6h or 24h pi and the treatment was continued until day 3 pi; lung viral loads were determined at day 4 post infection (Fig. 2A) When treatment was initiated at either 6 h or 24 h pi respectively a 3.0 log_10_ (P=0.002) and a 1.8 log_10_ (P=0.002) reduction in genome copies/mg lung tissue was observed as compared to the vehicle-treated infected hamsters (Fig. 2B). Likewise infectious virus titers in the lungs were reduced by respectively 3.1 (P=0.002) and 2.4 (P=0.002) log_10_ (Fig. 2C). In the 6h pi group a significant reduction (p=0.01) in the lung histopathology scores was observed as compared to the vehicle group (Fig. 2D). When treatment was initiated at 24h pi, an improvement in lung score was observed in only 2 out of 6 treated hamsters (Fig. 2D).

The combined Molnupiravir/Favipiravir treatment reduces viral transmission to contact hamsters

**Fig. 3:**
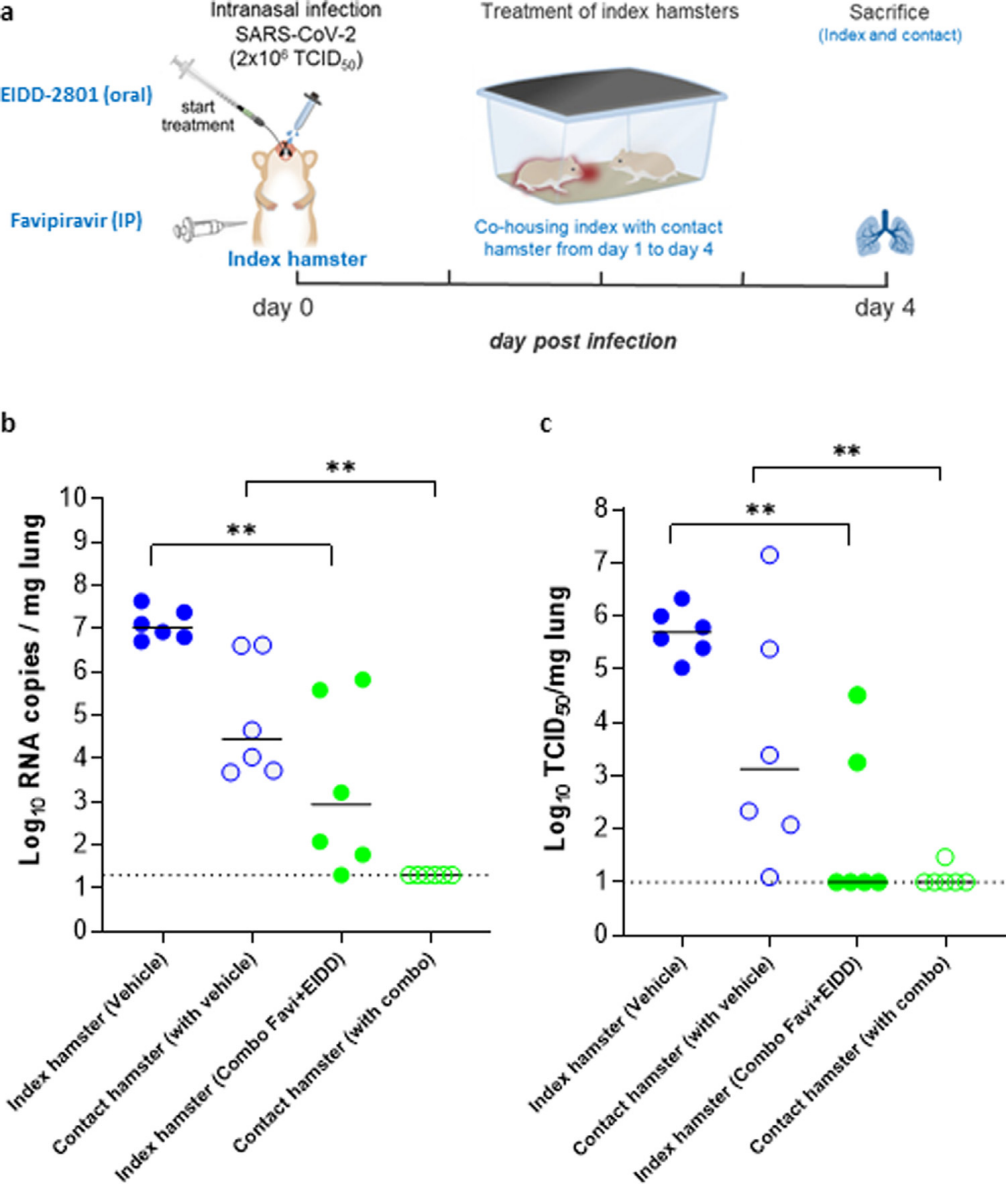
The effect of the combined Molnupiravir/Favipiravir treatment on viral transmission to contact hamsters. (a) Set-up of the study. (b) Viral RNA levels in the lungs of control (vehicle-treated), combination-treated (Favipiravir+EIDD-2801 at 300+150 mg/kg, BID, respectively) SARS-CoV-2−infected index hamsters (closed circles) and no-infected , non-treated contact hamsters (open circles) at day 4 post-infection (pi) are expressed as log_10_ SARS-CoV-2 RNA copies per mg lung tissue. Individual data and median values are presented. (c) Infectious viral loads in the lungs of control (vehicle-treated), combination-treated (Favipiravir+EIDD-2801) SARS-CoV-2−infected index hamsters and no-infected, non-treated contact hamsters at day 4 pi are expressed as log_10_ TCID_50_ per mg lung tissue. Individual data and median values are presented. Data were analyzed with the Mann−Whitney U test. **P < 0.01. Favi=Favipiravir, EIDD=EIDD-2801. The data are from a single experiment and with 6 animals per group.

Next, we studied whether treatment of hamsters with the Molnupiravir/Favipiravir combination would make them less infectious to untreated sentinels (Fig. 3A). To that end, index hamsters were infected with SARS-CoV-2 and treated from day 0 to day 3 with either vehicle or the combined therapy. Starting from day1 p.i. (6 hours after the first dose of the index hamsters), each of the index hamsters were co-housed in one cage with contact/sentinel hamsters. The co-housing was continued until 2.5 days after start of contact (Fig. 3A). The treatment of index hamsters with the combined Molnupiravir/Favipiravir therapy resulted in a marked reduction of viral RNA (4.1log_10_, P=0.002) and infectious viral loads (4.7 log_10_, P=0.002) in the lungs of the index animals with no infectious virus detectable in the lungs of 4 out of 6 hamsters (Fig. 3B/C), confirming the results presented in Fig. 1C. All sentinels that had been co-housed with vehicle-treated index hamsters had detectable viral RNA (Fig. 3B) and infectious virus loads in the lungs [ranging from 1.2x10^1^ to 1x10^7^ TCID_50_/mg lung tissue] (Fig. 3C). On the other hand, the viral RNA loads in the lungs of contact hamsters that were co-housed with the treated index hamsters were below the detection limit of the qRT-PCR assay (i.e. >20 genome copies/mg lung tissue, P=0.002 compared to the contact of vehicle-treated group) (Fig. 3B). Additionally, in 5 out of the 6 of these contact hamsters no infectious virus was detectable in the lungs (P=0.004 compared to the contact of vehicle-treated group). In the 6^th^ hamster only minutes amounts of infectious virus was detectable (30 TCID_50_/mg lung tissue) (Fig. 3C).

## 4 Discussion

Remdesivir (Veklury), is the first drug to have received FDA approval for use in hospitalised COVID-19 patients. The drug needs to be administrated intravenously which precludes its use in the early stages of the infection/disease or even a prophylactic use. We and others previously demonstrated that treatment of SARS-CoV-2-infected hamsters with high doses of Favipiravir largely reduces infectious virus titers in the animals and results as a consequence in a markedly improved lung pathology [4,21]. Favipiravir is currently being studied for the treatment of COVID-19 in clinical trials in several countries and was very recently reported to result in COVID-19 patients in a faster virological response, a shorter time to clinical symptoms and reduced mortality rates [5]. Also Molnupiravir has been reported to exert therapeutic and prophylactic activity against SARS-CoV-2 in several animal models [10], [11], [12]. Importantly, recent interim data from phase II clinical studies in COVID-19 patients, revealed a reduction in the time required to reach negative isolation of infectious virus from the nasopharyngeal swabs from participants with symptomatic SARS-CoV-2 infection [14]. Both Molnupiravir and Favipiravir have a high barrier to resistance, resistant variants have a loss in fitness and induce lethal viral mutagenesis [7,8]. Combination antiviral therapy of nucleoside analogs with favipiravir has been studied for treatment of other viral infections such as Lassa virus [22] and hantaviruses [23]. Treatment of Lassa virus-infected mice with a combination of suboptimal doses of ribavirin (80 mg/kg/day) and Favipiravir (150 mg/kg/day) resulted in significant prolongation of the survival duration and improved the survival rate compared to the single treatment groups [22]. We here studied the combined antiviral effect of both Favipiravir and Molnupiravir in a SARS-CoV-2 Syrian hamster infection model.

The combination of suboptimal doses of Molnupiravir and Favipiravir resulted in a marked antiviral activity in our hamster infection model. Infectious virus titers were reduced to undetectable levels in 10 out of 16 treated animals when the first dose was administered just before the infection. A median reduction of >4.5 log_10_ TCID_50_/mg lung tissue was achieved, which is markedly more pronounced than what could be expected from an additive activity of either Molnupiravir (1.3 log_10_) or Favipiravir (1.1 log_10_) when dosed alone. Even when start of treatment was delayed until 6h or 24h pi, a marked antiviral effect was achieved. Moreover, when sentinel animals were co-housed with treated infected hamsters, transmission of virus from the infected to the sentinel contact animals was nearly completely curbed. Consequently treatment of infected patients (e.g. soon after they have tested positive) may largely reduce the likelihood of transmission which could for example be important in post exposure household prophylaxis.

The pronounced efficacy of the combination may partially or even entirely be explained by the increased total mutation count (especially C-to-T mutations) in viral RNA collected from the lungs of combo-treated hamsters as compared to the single treatment groups.

In conclusion, the combination of Molnupiravir and Favipiravir (two oral drugs with a high barrier to resistance for which there is very recent initial evidence that they exert antiviral activity in COVID-19 patients), is particularly effective in the treatment of SARS-CoV-2 infections in hamsters and largely reduces transmission of the virus to uninfected contact sentinels. This efficacy may be explained by an enhanced accumulation of mutations as compared to monotherapy and may allow to use lower doses of the compounds in clinical settings. Our findings may lay the basis for the design of clinical studies to test the efficacy of the combination of Molnupiravir and Favipiravir in the treatment of COVID-19. Such combination could result in lowering the dose administered of both compounds

## 5 Contributors

All authors read and approved the final version of the manuscript. R.A., C.S.F., D.J. and J.N. designed the studies; R.A., C.S.F., S.J.F.K., X.Z., T.N.D.D., J.B., J.P., R.M. and L.L. performed the studies ; R.A., C.S.F., J.B, J.P. and B.W. analyzed data; D.J. and J.N. provided advice on the interpretation of data; R.A., C.S.F. and J.N. wrote the paper with input from co-authors; A.K.C. and S.D.J provided essential reagents; V.G. and E.H. provided and facilitated access to essential infrastructure; R.A., C.S.F., D.J., P.A., R.W. and J.N. supervised the study; L.V., L.C., P.L., J.N. and K.D. acquired funding. R.A., D.J. and J.N. have verified the underlying data.

## Declaration of Competing Interest

None to declare.

## Appendix Supplementary materials

Image, application 1

Image, application 2

Image, application 3
