# Supplementary material for: The combined treatment of Molnupiravir and Favipiravir results in a potentiation of antiviral efficacy in a SARS-CoV-2 hamster infection model
Source: eBioMedicine. 2021 Sep 24;72:103595. doi: 10.1016/j.ebiom.2021.103595 (PMC8461366; doi:10.1016/j.ebiom.2021.103595)
Supplement: Supplementary file 2 [file mmc2.pdf]

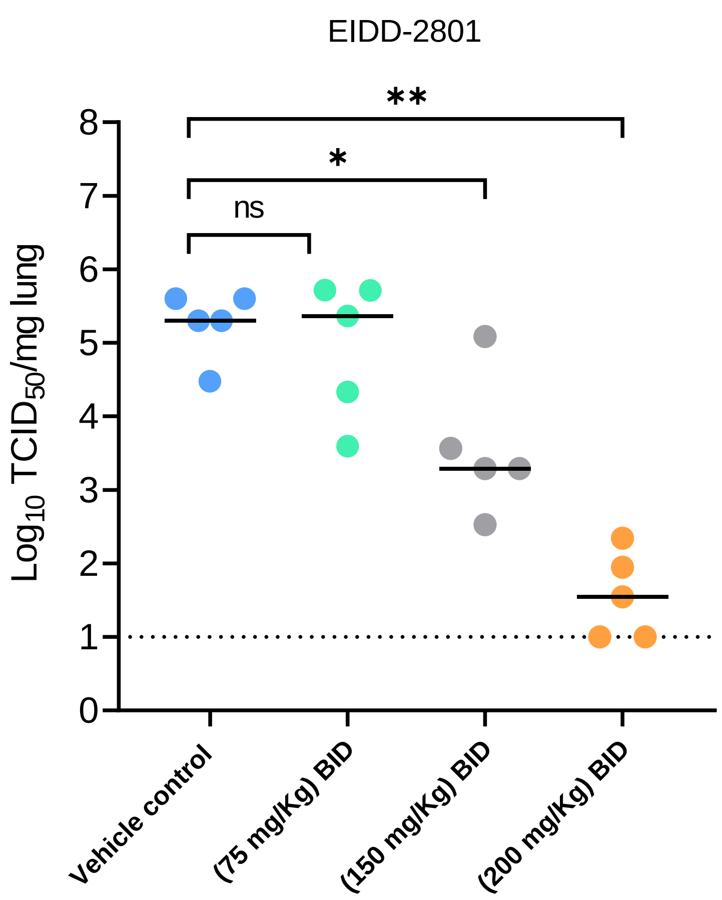

Fig. S1. Dose-response efficacy of Molnupiravir (EIDD-2801) against SARS-CoV-2 in a hamster infection model. Infectious viral loads in the lungs of control (vehicle-treated) and EIDD-2801-treated (at dose 75, 150 or 200 mg/kg twice daily for 4 consecutive days) SARS-CoV-2−infected hamsters at day 4 pi are expressed as $\log_{10}$ $TCID_{50}$ per mg lung tissue Individual data and median values are presented. Data were analyzed with the Mann−Whitney U test. *P < 0.05, **P < 0.01, ns=non-significant. The data are from a single experiment with 5 animals per group.

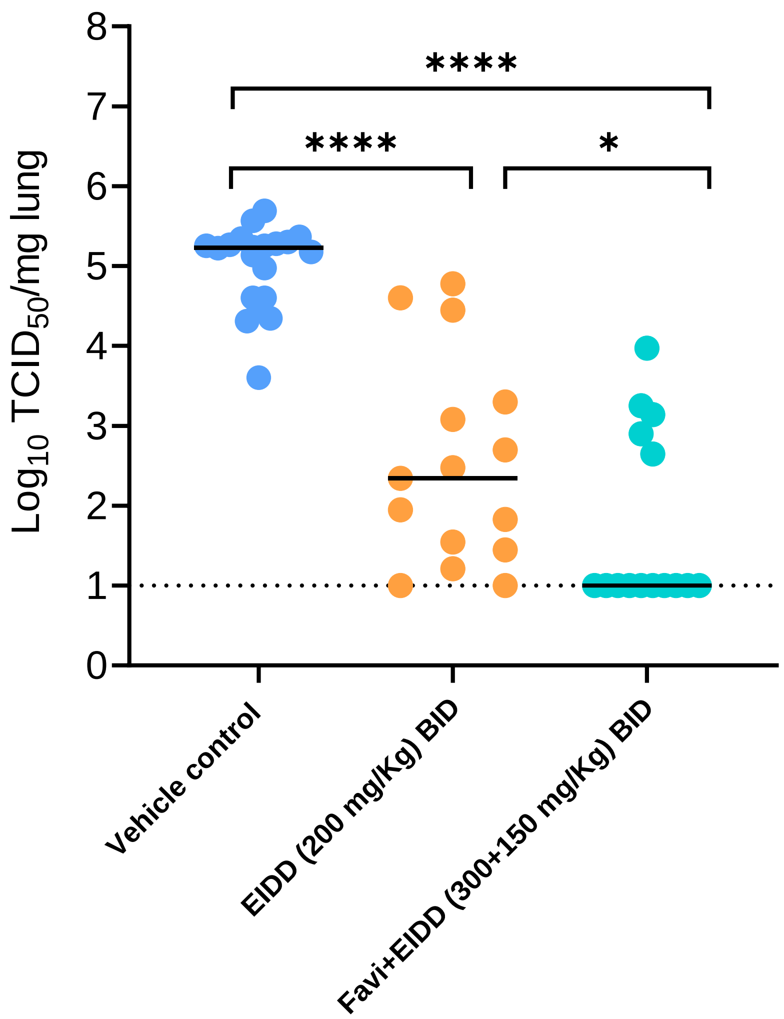

Fig. S2. Comparing the efficacy of the combined Favipiravir and Molnupiravir (EIDD-2801) (300+150 mg/kg, BID) treatment versus EIDD-2801 (200 mg/kg, BID) against SARS-CoV-2 in a hamster infection model. Infectious viral loads in the lungs of control (vehicle-treated), EIDD-2801-treated (200 mg/kg, BID) and combination-treated (Favipiravir+EIDD-2801) SARS-CoV-2−infected hamsters at day 4 pi are expressed as $\log_{10}$ $TCID_{50}$ per mg lung tissue. Individual data and median values are presented. Data were analyzed with the Mann−Whitney U test. *$P < 0.05$, ****$P < 0.0001$. EIDD=EIDD-2801. The data are from three independent experiments with 20, 15 and 15 animals for respectively the vehicle, EIDD-2801 (200 mg/kg, BID) and Favipiravir+EIDD-2801 (300+150 mg/kg, BID) condition.

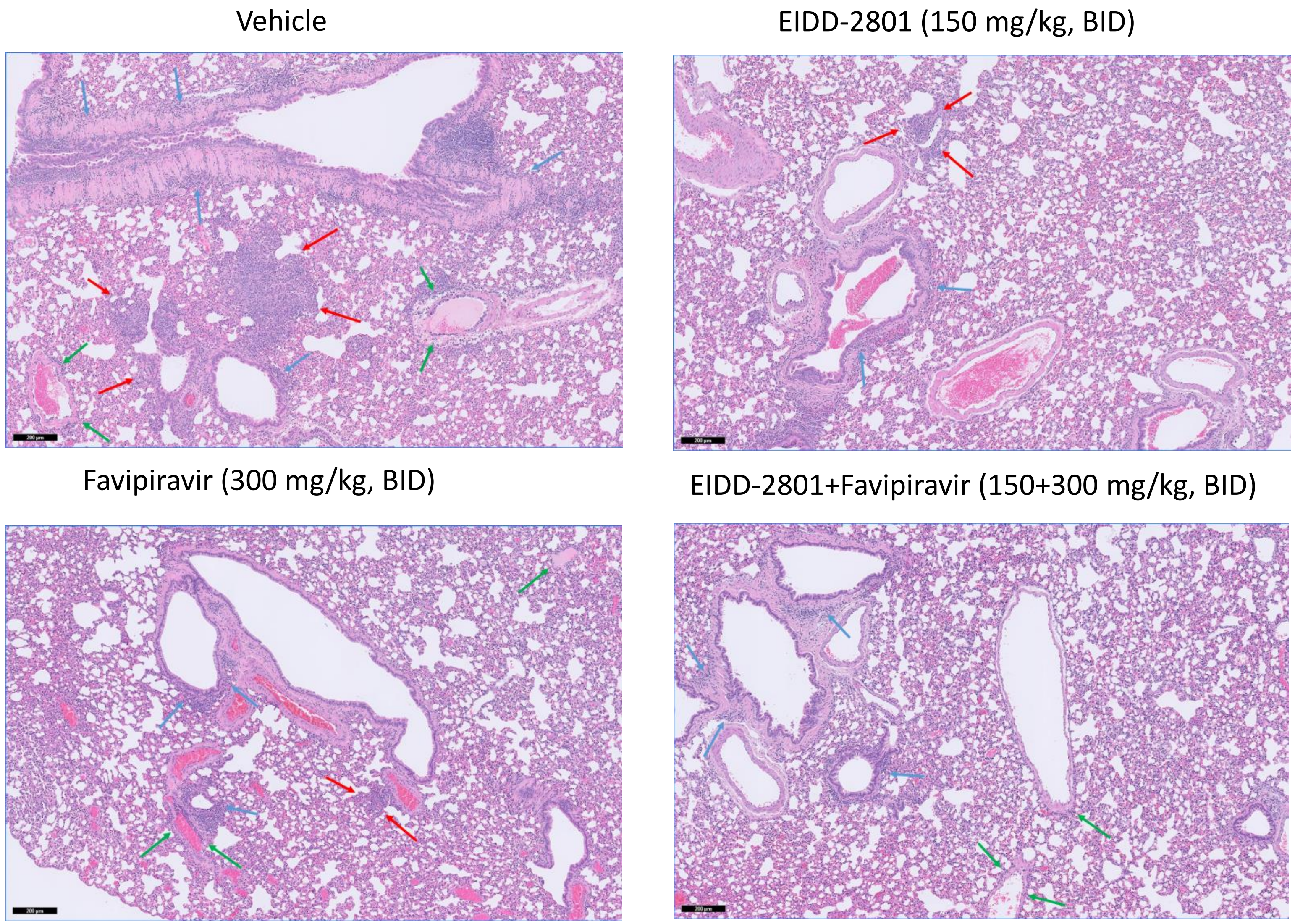

Fig. S3. Histopathology of lungs of Syrian hamsters treated with Molnupiravir (EIDD-2801), Favipiravir or a combination of both compounds. Representative H&E images of lungs of control (vehicle-treated), EIDD-2801-treated (150 mg/kg, BID), Favipiravir-treated (300 mg/kg, BID) and combination-treated (Favipiravir+EIDD-2801 at 300+150 mg/kg, BID, respectively) SARS-CoV-2−infected hamsters at day 4 post-infection (pi). The lungs of vehicle-treated infected hamsters show several regions with peri-bronchial inflammation (blue arrows), perivascular inflammation (green arrows) and bronchopneumonia (red arrows), whereas the lungs of compounds treated hamsters show improved lung pathology. No signs of bronchopneumonia are detected in the lungs from the combination-treated group. Scale bars, 200 µm.

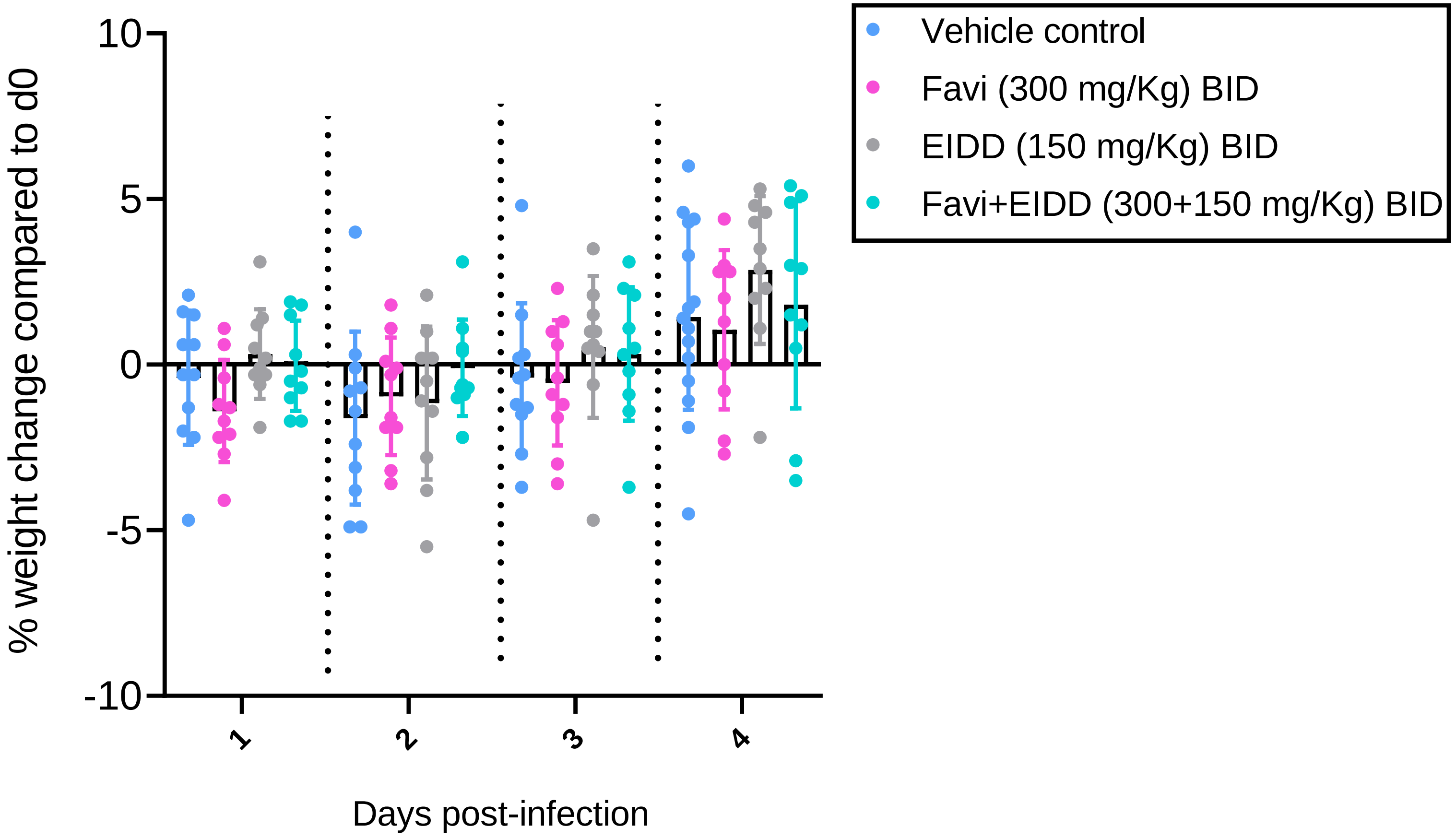

Fig. S4. Tolerability of combined treatment with favipiravir and EIDD-2801 in SARS-CoV-2-infected hamsters. Weight change at days 1-4 post-infection in percentage, normalized to the body weight at the time of infection (day zero, d0). Bars represent means ± SD.

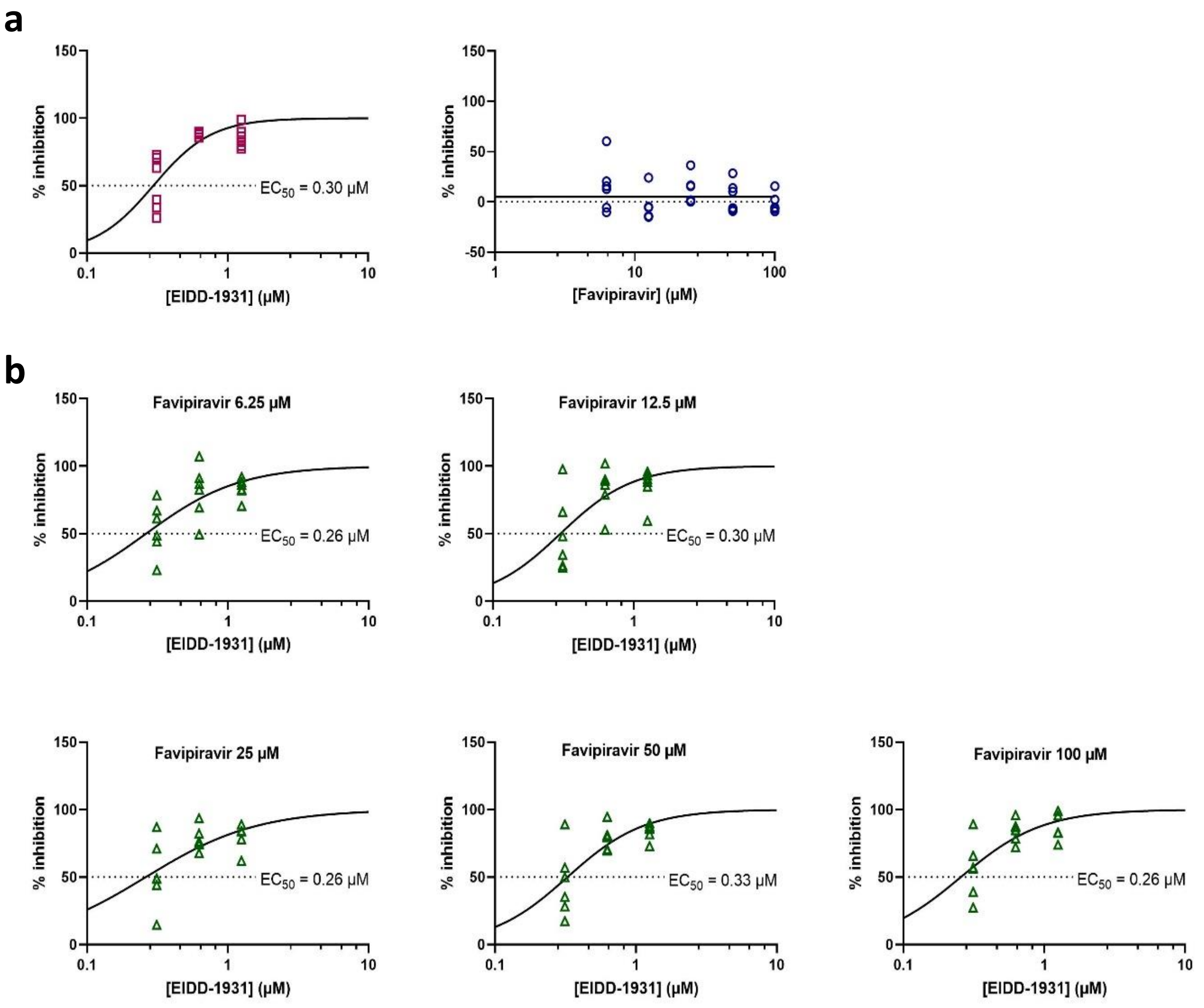

Fig. S5. Antiviral efficacy of EIDD-1931 and favipiravir in Vero E6-GFP cells. (A) Dose-dependent antiviral activity of either EIDD-1931 (open square) or favipiravir (open circle) monotherapy. Cells were seeded and pre-incubated with compounds for 24 hours before infected with SARS-CoV-2 at the MOI of 0.001 TCID50/cell. The inhibitory effect was determined by high-content imaging, based on the number of fluorescent pixels of GFP signals on day 4 post-infection (p.i.). (B) Percent inhibition of SARS-CoV-2 replication by EIDD-1931 and favipiravir combination therapy. Each graph represents the dose-response curve of the combined effects at the indicated concentration of favipiravir in presence of different concentrations of EIDD-1931.

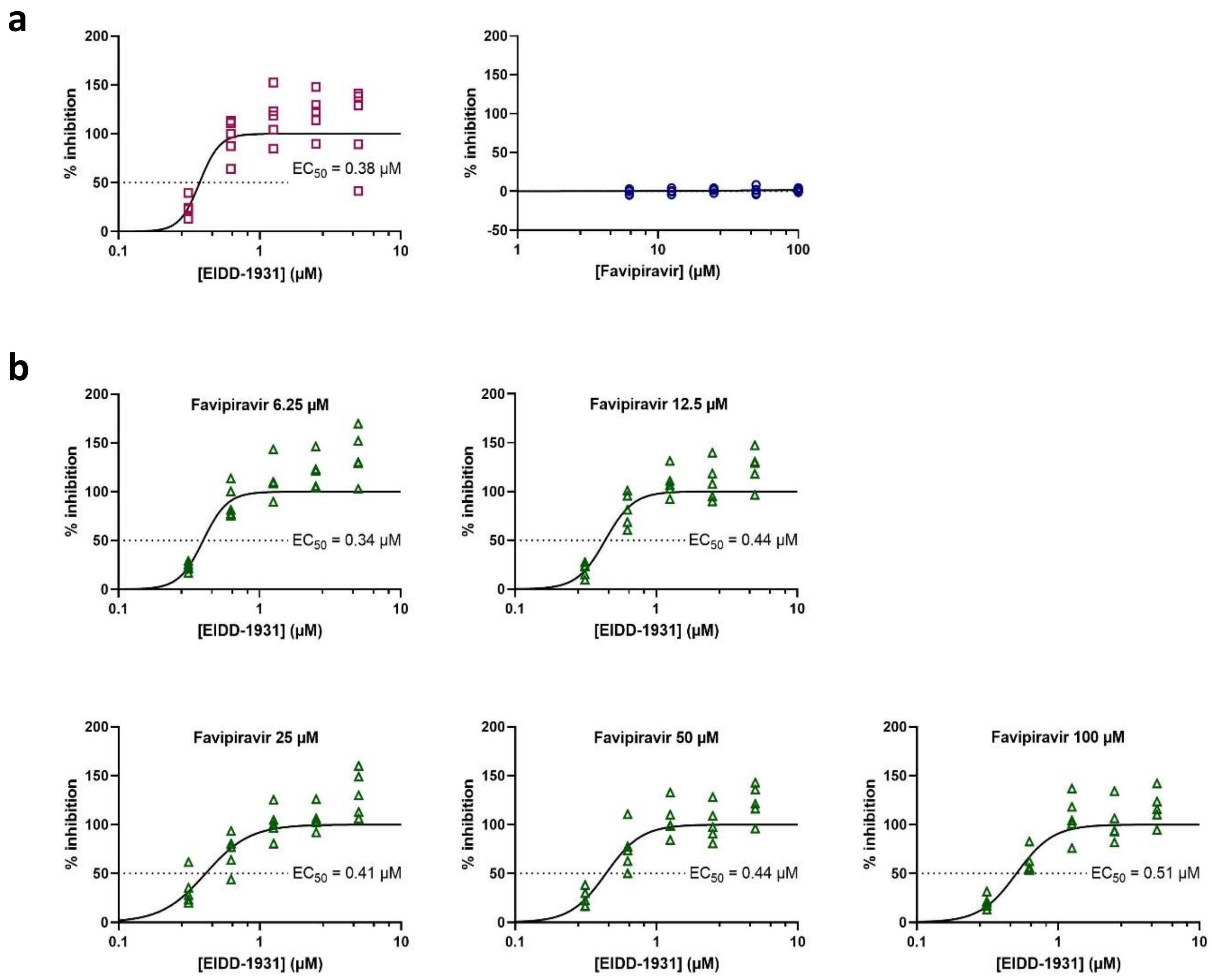

Fig. S6. Assessment of antiviral effects of EIDD-1931 and favipiravir in Huh-7 cells. (A) Dose-dependent antiviral activity of either EIDD-1931 (Open Square) or favipiravir (open circle) monotherapy. Cells were seeded and incubated overnight before exposed to compounds and infected with SARS-CoV-2 (MOI =0.002 TCID50/cell). The inhibitory effect was determined via MTS assay on day 4 p.i.. (B) Percent inhibition of SARS-CoV-2 replication by EIDD-1931 and favipiravir combination therapy. Each graph represents the dose-response curve of the combined effects at the indicated concentration of favipiravir in presence of different concentrations of EIDD-1931.

Supplementary Table S1: Histopathology scores for SARS-CoV-2 infected hamsters under different treatment conditions.

Treatment group	hamster ID	congestion	intralveolar hemorrhage	lymphoid follicles	apoptotic bodies in bronchus wall	necrotizing bronchiolitis	perivascular edema	bronchopneumonia	perivascular inflammation	peribronchial inflammation	vasculitis	Cumulative Score
**Vehicle control**	1	1			1			0.5	0.5	2		**5**
	2	1			1			2	0.5	2		**6.5**
	3	1			1			2	0.5	1		**5.5**
	4	1			1			2	0.5	1		**5.5**
	5	1			1			0.5	0.5	1		**4**
	6	1			1			0.5	0.5	1		**4**
	7	1			1			1	0.5	1		**4.5**
	8	1			1			1	0.5	1		**4.5**
	9	1			1			0.5	0.5	1		**4**
	10	1			1			1	1	1		**5**
	11	1			1			1	1	1		**5**
	12	1			1			1	0.5	2		**5.5**
	13	1			1			1	0.5	1		**4.5**
	14	1			1				1	1		**4**
	15	1			1			0.5	0.5	2		**5**
**EIDD-2801, 150 mg/kg**	16	1							0.5	0.5		**2**
	17	1						0.5	0.5	0.5		**2.5**
	18	1							0.5	0.5		**2**
	19	1			1			1		1		**4**
	20	1							0.5	0.5		**2**
	21	1		1	1			1	1	1		**5**
	22	1			1			0.5	0.5	1		**4**
	23	1			1			0.5		0.5		**3**
	24	1			1			2	0.5	1		**5.5**
	25	1						0.5	0.5	0.5		**2.5**
**Favipiravir, 300 mg/kg**	26	1			1			0.5	0.5	1		**4**
	27	1	1						1			**3**
	28	1	1		1			2	1	1	0.5	**7.5**
	29	1	1		1			2	1	0.5	0.5	**7**
	30	1			1			0.5	0.5	1		**4**
	31	1			1			1	0.5	1		**4.5**
	32	1			1					1		**3**
	33	1			1			1	0.5	1	0.5	**5**
	34	1			1			1	1	1		**5**
	35	1			1			0.5	0.5	0.5		**3.5**
**Combination Favipiravir+ EIDD-2801 (300+150 mg/kg)**	36	1		1					0.5			**1.5**
	37	1							0.5	0.5		**2**
	38	1			0.5				0.5	0.5		**2.5**
	39	1							0.5			**1.5**
	40	1			0.5				0.5	0.5		**2.5**
	41	1										**1**
	42	1							0.5	0.5		**2**
	43	1			1			0.5	0.5	1		**4**
	44	1			1					1		**3**
	45	1			1			0.5	0.5	1		**4**

Supplementary Table S2: Pharmacokinetics of favipiravir and Molunpiravir (EIDD-2801) in plasma from SARS-CoV-2-infected hamsters

Sample number	Plasma concentration, 16h after the last dose (nM)			
	Single treatment		Combination treatment	
	EIDD-2801 (150 mg/kg)	Favipiravir (300 mg/kg)	EIDD-2801 (150 mg/kg)	Favipiravir (300 mg/kg)
1	52	11081	111	3806
2	51	9191	96	3376
3	44	5820	100	4930
4	40	8352	90	6213
5	28	6930	82	12767
6	30	3054	59	3897
7	27	3183	71	4876
8	30	4160	61	4633
9	26	3431	55	4095
10	40	4450	59	4044

Supplementary Table S3: Mutation count per SARS-CoV-2 genome as determined by deep sequencing of viral RNA isolated from the lungs of infected hamsters under different treatment conditions.

Treatment	Mutation count per SARS-CoV-2 genome											
	AC	AG	AT	CA	CG	CT	GA	GC	GT	TA	TC	TG
Vehicle	1	2	1	0	0	5	0	0	0	0	2	0
Vehicle	0	5	2	2	0	8	1	0	0	0	0	0
Vehicle	0	1	0	2	0	3	0	0	1	0	3	0
Vehicle	1	0	1	1	0	6	0	0	0	0	1	1
Vehicle	2	2	0	0	1	8	0	0	1	0	6	0
Favipiravir-300 mg/kg	0	1	0	0	0	12	19	0	0	0	2	0
Favipiravir-300 mg/kg	0	0	1	0	0	7	11	0	0	0	2	0
Favipiravir-300 mg/kg	0	2	0	0	0	7	16	0	0	0	1	1
Favipiravir-300 mg/kg	2	3	0	1	0	27	40	0	0	0	6	0
Favipiravir-300 mg/kg	0	1	2	1	0	17	17	1	1	0	4	0
EIDD-2801-150 mg/kg	1	8	0	1	0	27	28	0	0	0	3	1
EIDD-2801-150 mg/kg	1	13	1	0	0	39	33	2	1	0	13	1
Combo EIDD+Favi-150+300 mg/kg	1	6	5	1	0	74	52	1	2	0	21	1
Combo EIDD+Favi-150+300 mg/kg	0	10	2	1	0	61	47	0	1	0	19	2
