# Supplementary material for: The combined treatment of Molnupiravir and Favipiravir results in a potentiation of antiviral efficacy in a SARS-CoV-2 hamster infection model
Source: eBioMedicine. 2021 Sep 24;72:103595. doi: 10.1016/j.ebiom.2021.103595 (PMC8461366; doi:10.1016/j.ebiom.2021.103595)
Supplement: Supplementary file 3 [file mmc3.pdf]

EBIOM-D-21-01724 paper

REPLY REPLY ALL FORWARD •••

Mark as read

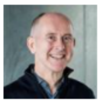

Patrick Augustijns

Tue 8/24/2021 11:42 AM

To: ☐ Rana Abdelnabi;

I, Patrick Augustijns, agree on including my name as co-author in the final version of the **EBIOM-D-21-01724**manuscript

EBIOM-D-21-01724 paper

REPLY REPLY ALL FORWARD •••

Mark as read

Raf Mols

Fri 8/27/2021 2:56 PM

To: ☐ Rana Abdelnabi;

Me, Raf Mols, agree on including my name as co-authors in the final version of the **EBIOM-D-21-01724**manuscript

Me, Rana Abdelnabi, Agree on including Raf Mols and Patrick Augustins to the final version of EBIOM-D-21-01724.

Yours sincerely,

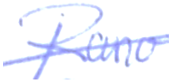

Rana Abdelnabi, PhD

Urgent: EBIOM-D-21-01724

REPLY REPLY ALL FORWARD •••

Mark as read

Shi-Yan Caroline Foo

Wed 8/25/2021 10:59 AM

To: ☐ Rana Abdelnabi;

Hello Rana,

Here below:

"Me, Caroline S. Foo, agree on including Raf Mols and Patrick Augustijns as co-authors in the final version of the **EBIOM-D-21-01724**manuscript"

Best,

Caroline

Urgent: EBIOM-D-21-01724

REPLY REPLY ALL FORWARD •••

Mark as unread

Lana Langendries
Tue 8/24/2021 10:54 AM

To: ☐ Rana Abdelnabi;

Congratulations Rana!

Dear Editor,

Me, Lana Langendries agree on including Raf Mols and Patrick Augustijns as co-authors in the final version of the EBIOM-D-21-01724 manuscript.

Best,

Lana Langendries
PhD student

REPLY REPLY ALL FORWARD •••

Mark as unread

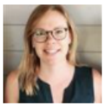

Laura Vangeel
Tue 8/24/2021 10:45 AM

Show all 18 recipients

To: ☐ Rana Abdelnabi; Shi-Yan Caroline Foo; Xin Zhang; ☐ Lana Langendries; ☐ Dirk Jochmans; ☐ Nguyen Dan Thuc Do; Suzanne Kaptein; ...

Dear Editor,
Dear Rana

Me, Laura Vangeel, agree on including Raf Mols and Patrick Augustijns as co-authors in the final version of the EBIOM-D-21-01724 manuscript.

Best,
Laura

REPLY REPLY ALL FORWARD •••

Mark as unread

Pang, Juanita <juanita.pang.16@ucl.ac.uk>
Tue 8/24/2021 10:30 AM

Show all 18 recipients

To: ☐ Breuer, Judith <j.breuer@ucl.ac.uk>; ☐ Rana Abdelnabi; Shi-Yan Caroline Foo; Xin Zhang; ☐ Lana Langendries; ☐ Dirk Jochmans; ☐ Nguyen Dan Thuc Do; ...

Thanks Rana!

Me, Juanita Pang, agree on including Raf Mols and Patrick Augustijns as co-authors in the final version of the **EBIOM-D-21-01724** manuscript

REPLY REPLY ALL FORWARD •••

Mark as unread

Breuer, Judith <j.breuer@ucl.ac.uk>
Tue 8/24/2021 10:28 AM

Show all 18 recipients

To: ☐ Rana Abdelnabi; Shi-Yan Caroline Foo; Xin Zhang; ☐ Lana Langendries; ☐ Dirk Jochmans; ☐ Nguyen Dan Thuc Do; Suzanne Kaptein; ...

Well done Rana and co!

Judith Breuer agrees to including Raf Mols and Patrick Augustijns as co-authors in the final version of the **EBIOM-D-21-01724** manuscript"

REPLY REPLY ALL FORWARD •••

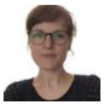

Lotte Coelmont

Tue 8/24/2021 10:12 AM

Mark as unread

To: ☐ Rana Abdelnabi;

Dear Editor, Dear Dr. Abdelnabi,

"Me, Lotte Coelmont, agree on including Raf Mols and Patrick Augustijns as co-authors in the final version of the **EBIOM-D-21-01724** manuscript"

Yours Sincerely,

Lotte Coelmont

REPLY REPLY ALL FORWARD •••

Birgit Weynand <birgit.weynand@uzleuven.be>

Tue 8/24/2021 10:05 AM

Mark as unread

To: ☐ Rana Abdelnabi;

Dear Rana,
Indeed very good news.
Me, Weynand Birgit, agree on including Raf Mols and Patrick Augustijns as co-authors in the final version of the **EBIOM-D-21-01724** manuscript

Best regards
Birgit

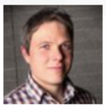

Kai Dallmeier

Tue 8/24/2021 10:01 AM

Mark as unread

Show all 18 recipients

To: ☐ Rana Abdelnabi; Shi-Yan Caroline Foo; Xin Zhang; ☐ Lana Langendries; ☐ Dirk Jochmans; ☐ Nguyen Dan Thuc Do; Suzanne Kaptein; ...

• You replied on 8/24/2021 10:02 AM.

Dear Editor,
Dear Rana

Me, Kai Dallmeier, agree on including Raf Mols and Patrick Augustijns as co-authors in the final version of the **EBIOM-D-21-01724** manuscript.

Kind regards

Kai Dallmeier, PhD (Dr. rer. nat.)

Valentijn Vergote
Tue 8/24/2021 9:47 AM

Mark as unread

To: ☐ Rana Abdelnabi;

You replied on 8/24/2021 9:49 AM.

Congrats Rana!
Will it be a seperate email or this email?
All the best!

Me, Valentijn Vergote, agree on including Raf Mols and Patrick Augustijns as co-authors in the final version of the **EBIOM-D-21-01724** manuscript

REPLY REPLY ALL FORWARD •••

Nguyen Dan Thuc Do
Tue 8/24/2021 9:46 AM

Mark as unread

To: ☐ Rana Abdelnabi;

Hi Rana,

Congratulations!!! You can find my acceptance statement as below.

"I, Thuc Nguyen Dan Do, agree on including Raf Mols and Patrick Augustijns as co-authors in the final version of the **EBIOM-D-21-01724** manuscript"

Cheers,
Thuc

REPLY REPLY ALL FORWARD •••

Pieter Leyssen
Tue 8/24/2021 9:40 AM

Mark as unread

To: ☐ Rana Abdelnabi;

You replied on 8/24/2021 9:41 AM.

Me, Pieter Leyssen, agrees on including Raf Mols and Patrick Augustijns as co-authors in the final version of the **EBIOM-D-21-01724** manuscript

REPLY REPLY ALL FORWARD •••

Dirk Jochmans
Tue 8/24/2021 9:39 AM

Mark as unread

To: ☐ Rana Abdelnabi;

Hey Rana

I, Dirk Jochmans, agree on including Raf Mols and Patrick Augustijns as co-authors in the final version of the EBIOM-D-21-01724 manuscript.

Regards,
Dirk J

Urgent: EBIOM-D-21-01724

REPLY REPLY ALL FORWARD •••

Mark as read

Elisabeth Heylen
Tue 8/24/2021 1:24 PM

Show all 18 recipients

To: Laura Vangeel; Rana Abdelnabi; Shi-Yan Caroline Foo; Xin Zhang; Lana Langendries; Dirk Jochmans; Nguyen Dan Thuc Do; ...

Dear Editor,
Dear Rana

Me, Elisabeth Heylen, agree on including Raf Mols and Patrick Augustijns as co-authors in the final version of the EBIOM-D-21-01724 manuscript.

Regards
Elisabeth

Urgent: EBIOM-D-21-01724

REPLY REPLY ALL FORWARD •••

Mark as read

Xin Zhang
Tue 8/24/2021 2:12 PM

Show all 18 recipients

To: Rana Abdelnabi; Shi-Yan Caroline Foo; Lana Langendries; Dirk Jochmans; Nguyen Dan Thuc Do; Suzanne Kaptein; Laura Vangeel; ...

Dear Editor,
Dear Rana,

Me, Xin Zhang, agree on including Raf Mols and Patrick Augustijns as co-authors in the final version of the **EBIOM-D-21-01724** manuscript.

Best regards,
Xin Zhang

REPLY REPLY ALL FORWARD •••

Mark as unread

Suzanne Kaptein
Tue 8/24/2021 3:18 PM

To: Rana Abdelnabi;

You replied on 8/24/2021 3:18 PM.

Hi Rana,

See below for confirmation of adding the two co-authors to the final version of the manuscript.

Best,
Suzanne

Dear Editor,
Dear Rana

Me, Suzanne J.F. Kaptein, agree on including Raf Mols and Patrick Augustijns as co-authors in the final version of the EBIOM-D-21-01724 manuscript.

Kind regards,
Suzanne

Urgent: EBIOM-D-21-01724

REPLY REPLY ALL FORWARD •••

Steven De Jonghe
Wed 8/25/2021 9:25 AM

Mark as read

Show all 18 recipients

To: Rana Abdelnabi; Shi-Yan Caroline Foo; Xin Zhang; Lana Langendries; Dirk Jochmans; Nguyen Dan Thuc Do; Suzanne Kaptein; ...

Dear Editor,
Dear Rana,

"Me, Steven De Jonghe, agree on including Raf Mols and Patrick Augustijns as co-authors in the final version of the EBIOM-D-21-01724 manuscript"

Kind regards
Steven

REPLY REPLY ALL FORWARD •••

Arnab Chatterjee <achatterjee@scripps.edu>
Tue 8/24/2021 7:23 PM

Mark as unread

Show all 18 recipients

To: Xin Zhang; Rana Abdelnabi; Shi-Yan Caroline Foo; Lana Langendries; Dirk Jochmans; Nguyen Dan Thuc Do; Suzanne Kaptein; ...

You replied on 8/24/2021 8:28 PM.

Me, Arnab Chatterjee, agree on including Raf Mols and Patrick Augustijns as co-authors in the final version of the EBIOM-D-21-01724 manuscript

Urgent: EBIOM-D-21-01724

REPLY REPLY ALL FORWARD •••

Williams, Rachel <rachel.williams@ucl.ac.uk>
Thu 8/26/2021 4:51 PM

Mark as read

To: Rana Abdelnabi;

Dear Rana,

Apologies for the delay in replying but I have been away.

I, Rachel Williams, agree on including Raf Mols and Patrick Augustijns as co-authors in the final version of the **EBIOM-D-21-01724** manuscript.

BW,

Rachel
